# Evaluation of AI tools for triage and risk stratification in emergency medicine

**DOI:** 10.6026/973206300213804

**Published:** 2025-10-31

**Authors:** Nikhil Paul, Parth Jani, Niyati Pandya, Amrit Podder, Mukul Singh, Carlos Andres Chango Rodriguez

**Affiliations:** 1 Department of Emergency Medicine, Kasturba Medical College Mangalore, Manipal Academy of Higher Education, Manipal, India; 2 Department of General Medicine, Government Medical College, Bhavnagar, Gujarat, India; 3 Department of Anaesthesiology, All India Institute of Medical Sciences, Rajkot, Gujarat, India; 4 Department of Physiology, Teerthanker Mahaveer Medical College & Research Centre, Teerthanker Mahaveer University, Moradabad, Uttar Pradesh, India; 5 Department of General Surgery, All India Institute of Medical Sciences, Gorakhpur, Uttar Pradesh, India; 6 Department of Medicine, Physician Armed Forces Hospital, Quito, Ecuador, South America

**Keywords:** Accuracy, AI-assisted triage, emergency medicine, risk stratification, triage

## Abstract

The effectiveness of AI tools in triage and risk stratification in emergency medicine by comparing outcomes between AI-assisted triage and traditional triage methods is of interest. Hence, a total of 300 patients were enrolled, with 150 in each group, over 14 days. Results showed that the AI-assisted triage group had significantly reduced time to treatment, higher accuracy in triage decisions and more efficient resource allocation. Patient outcomes and length of stay were similar across both groups. Data shows that AI tools can enhance triage efficiency without compromising patient care.

## Background:

Artificial Intelligence (AI) has proven to be a game-changer in different spheres, including in healthcare [1]. When it comes to any emergency care where time and the ability to make a decision are crucial, AI tools can potentially improve triage protocols and risk stratification so that no patient will go without the necessary care [2]. Due to the increased need for emergency care in recent years, which is highly based on the complexity of patients presented, it has become a busy problem in prioritising care. The process of triage, an evaluation of patients by their medical needs to ascertain the urgency of their medical requirements, is conventionally made based on the judgment of a human. It is therefore prone to error and may also have delays [3]. However, AI tools can analyse this type of data in large volumes at a very rapid rate and with high accuracy, which may enable faster and more accurate triage decisions [4]. In AI, different sorts of data, such as vital signs, medical history and real-time diagnostics, can be incorporated by the system to determine an objective patient severity [5]. Such systems employ the application of algorithms to serve the patients based on several aspects, including the likelihood of deterioration, the need for prompt intervention and the possibility of positive outcomes. AI can also be used to predict patient outcomes in risk stratification and behaviour since it can analyse even their patterns that may not be obvious to healthcare providers [6]. This may enable optimal use of resources, shorter wait times and high-risk patients identified and treated first. Nevertheless, even though the potential of having AI in emergency medicine is excellent, its introduction creates a series of issues, such as questioning the accuracy and reliability of AI systems, the risk of insufficient training of medical practitioners and the ethics of letting the AI make the put-or-take decision in life and death [7]. Also, it is necessary to develop evidence-based validation of such tools in various clinical conditions to achieve their effectiveness [8]. Therefore, it is of interest to determine the efficacy, accuracy and practicality of AI tools in triage and risk stratification in emergency medicine.

## Methodology:

A prospective cohort study implementation was selected to determine the utility of AI tools in emergency medicine as part of the triage and risk stratification. Outcomes in triaging with AI-assisted tools used in emergency departments (EDs) versus traditional triaging were compared over a 14-day monitoring study period. Speed, accuracy and effectiveness of triage decisions and risk stratification were the primary targets of choice. With monitoring clinical data related to time to treatment, the level of appropriateness of prioritising patients, distributing resources and ultimately patient outcomes, the study sought to indicate any advantageous effect that AI tools could have on the triage process. The research included 300 patients in a sample (150 patients in the AI-assisted triage group and 150 patients in the traditional triage group). This sample size was considered adequate to observe significant differences in the results with a moderate-sized effect, statistical power of 80% and alpha of 0.05. Patients were randomly divided into two groups: AI-assisted triage, during which patient information data (including vital signs, medical history and lab tests) were evaluated with the help of AI tools suggesting the triage decisions or the traditional triage in which the healthcare providers utilized their clinical judgment and existing triage systems, such as the Emergency Severity Index, to assess and prioritize patients. The interval of the study was established within 14 days, during which it was required to observe consistent patient flow and capture the diversity of emergent cases. This period provided that the gathering of data on the effectiveness of triage was strong, indicating normal functions of emergency departments. The collected information was based on electronic health records (EHRs) and the hospital's database and only the essential variables were provided, including patient demographics, triage decision and classification, time received, resource consumption, patient outcomes, such as hospitalisation, mortality and complications. The accuracy of the triage decisions was the primary study outcome. It was measured by comparing the recommendations made with the help of an AI tool with the final clinical outcomes of the assessed patient, i.e. the accuracy of the triage priorities. Secondary outcomes encompassed time to initial treatment, ED length of stay, patient outcome (e.g., mortality, complications) and resource utilisation (e.g. the number of diagnostic tests, health care staff resources). Comparisons were done using chi-square tests, independent t-tests and ANOVA on the two groups, with chi-square tests on categorical variables and independent t-tests and ANOVA on continuous variables. The statistical significance was determined to be p < 0.05. The given methodology has contributed to a concise assessment of how AI tools can influence triage and risk stratification in emergency surgical care and supplied firsthand knowledge of the practice of applying them and their possible contribution to the enhancement of patient care.

## Results:

The study aimed to evaluate the effectiveness of AI tools in triage and risk stratification in emergency medicine by comparing outcomes between two groups: those receiving AI-assisted triage and those using traditional triage methods. Data was collected over 14 days and a total of 300 patients were enrolled, with 150 patients in each group. The primary and secondary outcomes were analysed and the findings are presented below. The study included a total of 300 patients, with 150 patients assigned to the AI-assisted triage group and 150 to the traditional triage group. The analysis focused on comparing both groups in terms of demographic characteristics, time to treatment, triage accuracy, resource utilization, patient outcomes and length of stay in the emergency department over 14 days. In terms of demographics, both groups were comparable. The average age of patients in the AI-assisted group was 43.2 years, while the traditional group had a mean age of 42.8 years. The gender distribution was nearly equal in both groups, with males comprising slightly over half of the sample in each group. Additionally, the proportion of patients with prior medical conditions was similar between the two groups, indicating that the random allocation process was successful and both cohorts were balanced at baseline. Table 1 (see PDF) shows the demographic characteristics of the two groups. Both groups were similar in terms of age, gender and medical history, ensuring that the randomisation process was effective. When analysing time to initial treatment, the AI-assisted triage group showed a significantly faster response time. On average, patients in this group were treated within 12.5 minutes, compared to 18.2 minutes in the traditional triage group. This difference was statistically significant (p < 0.001), suggesting that AI tools helped expedite clinical decision-making and improved patient flow. Regarding triage accuracy, the AI-assisted triage group demonstrated higher accuracy in correctly classifying the severity of patient conditions. The AI group achieved an 89.3% accuracy rate, while the traditional group had a lower rate of 74.7%. This difference was also statistically significant (p < 0.001), indicating that AI tools enhanced the precision of triage decisions. Table 2 (see PDF) presents the time to initial treatment for both groups. The AI-assisted triage group had a significantly shorter time to treatment compared to the traditional triage group, highlighting the efficiency of the AI system in prioritising patients. In terms of resource allocation, the AI-assisted group used fewer diagnostic tests and required less staff time per patient. Patients triaged with AI tools underwent an average of 1.4 diagnostic tests, compared to 2.1 in the traditional group. Similarly, staff spent an average of 4.3 hours per patient in the AI group, while the conventional group required 5.2 hours. Both results were statistically significant, demonstrating more efficient use of hospital resources when AI tools were employed. Table 3 (see PDF) shows the accuracy of triage decisions between the two groups. The AI-assisted triage group had a higher accuracy in correctly identifying the severity of patient conditions, leading to more appropriate resource allocation. Patient outcomes, including hospital admissions, complications and mortality, showed no significant differences between the groups. Hospital admission rates were 32.0% in the AI group and 34.7% in the traditional group. Complication rates were 6.7% and 7.3%, respectively, while mortality was equal at 1.3% in both groups. These findings suggest that the use of AI did not negatively impact patient safety or outcomes. Table 4 (see PDF) presents the differences in resource utilisation between the two groups. The AI-assisted triage group showed a more efficient use of resources, with fewer unnecessary tests and diagnostic procedures. Finally, the average length of stay in the emergency department was significantly shorter for patients in the AI-assisted triage group. They spent an average of 3.8 hours in the ED compared to 4.5 hours for patients triaged traditionally (p < 0.001)-this reduction in ED time points to increased operational efficiency and potentially less crowding. Table 5 (see PDF) shows patient outcomes in terms of hospital admission, complications and mortality. There was no significant difference in patient outcomes between the two groups, suggesting that the AI-assisted triage system did not adversely affect patient care. Table 6 (see PDF) presents the length of stay in the emergency department (ED) for both groups. The AI-assisted triage group had a shorter length of stay, which is indicative of more efficient patient flow and decision-making. Overall, the results of this study demonstrated that AI-assisted triage significantly improved the speed and accuracy of emergency medical assessments, reduced unnecessary resource use and shortened ED length of stay, all without compromising patient safety. These findings support the integration of AI tools into emergency department workflows to optimise triage and risk stratification processes.

## Discussion:

This study reproduces previous studies that looked into the field of AI in emergency medicine. Research by Marchiori *et al.* (2021) [9] has suggested that AI-informed systems had an enhanced level of accuracy to the point where a triage decision surpassed that of the human triage nurse by more than 20 per cent. In the same way, the group responding under AI-based triage demonstrated an accuracy rate of 89.3%, which is considerably better than the traditional triage, which had an accuracy rate of 74.7%. This justifies the belief that AI tools may help supplement the correctness of initial assessments of patients, which is essential to prioritise patients and allocate resources. Regarding the time to treatment, the AI group in this study reduced the response time between and within individual subjects and the median was 12.5 minutes, which was significantly shorter than the traditional group, where it was 18.2 minutes. Some of these previous studies, like a study by Townsend *et al.* (2023) [10], found that there had also been similar differences in the wait times with the support of AI triage, in that patients who were triaged through AI had less time taken for the decision-making process and a delay in receiving treatment. These findings are similar to our findings, supporting the prospects of AI to accelerate emergency care and enhance overall patient flow in overcrowded EDs. Another significant discovery of this research study is the decrease in resource use. Using AI triage reduced the number of diagnostic tests (1.4 tests per patient versus 2.1) and staff time per patient (4.3 versus 5.2 hours). This observation correlates with similar studies conducted by Li *et al.* (2021) [11], which showed that the AI triage system has led to a more effective utilisation of available resources, thus reducing healthcare staff workload. AI will help reduce decision paralysis by acting as a first line of help in making an initial assessment of patients and helping to prioritise more critical cases so that the medical professionals have time to treat more urgent patients. Although these actions can be described as positive, there was no significant distinction between the rates of patient outcomes in the two groups, hospital admissions, or adverse events. This aligns with a study by Ahmed Abdalhalim *et al.* (2025) [12] that revealed that. In contrast, triage systems assisted by AI were able to bolster operational efficiency. They had no impact on clinical performance, i.e., patient safety or mortality. The fact that this study did not reveal any adverse effects indicates that AI can be deployed in clinical practice safely and without depreciating patient care.

## Conclusion:

A process of triage facilitated by AI enhances the speed, precision and efficiency of emergency procedures without inflicting adverse patient outcomes is shown. The combination of AI tools will help to improve efficiency in resource utilisation and decrease the congestion in ED. Long-term effects and implications on healthcare systems beyond small scale are yet to be determined in further research.
